# An Explanation of the Underlying Mechanisms for the In Vitro and In Vivo Antiurolithic Activity of* Glechoma longituba*

**DOI:** 10.1155/2016/3134919

**Published:** 2016-10-20

**Authors:** Qiang Liang, Xiaoran Li, Wangning Zhou, Yu Su, Shenbao He, Shuanglei Cheng, Jianzhong Lu, Wenjuan Cao, Yuke Yan, Xiaxia Pei, Jin Qi, Guangli Xu, Zhongjin Yue

**Affiliations:** ^1^Department of Urology, Institute of Urology, Gansu Nephro-Urological Clinical Center, Key Laboratory of Urological Diseases in Gansu Province, The Second Hospital of Lanzhou University, Lanzhou, Gansu 730030, China; ^2^Department of Neurosurgery, The Neurosurgery Clinical Medical Center, The Second Hospital of Lanzhou University, Lanzhou, Gansu 730030, China; ^3^Department of Urology, First Affiliated Hospital of Anhui Medical University and Institute of Urology, Anhui Medical University, Hefei, Anhui, China; ^4^First Clinical Medical College of Lanzhou University, Lanzhou, Gansu 730000, China; ^5^Department of General Surgery, Lanzhou University Second Hospital, Lanzhou 730030, China; ^6^Orthopaedics Key Laboratory of Gansu Province, Department of Orthopaedics, The Second Hospital of Lanzhou University, Lanzhou, Gansu, China; ^7^Department of Cardiology, Lanzhou University Second Hospital, Lanzhou 730030, China

## Abstract

*Purpose*. To use in vitro and in vivo models to evaluate* Glechoma longituba* extract to provide scientific evidence for this extract's antiurolithic activity.* Materials and Methods*. Potassium citrate was used as a positive control group. Oxidative stress (OS) markers and the expression of osteopontin (OPN) and kidney injury molecule-1 (KIM-1) were measured to assess the protective effects of* Glechoma longituba*. Multiple urolithiasis-related biochemical parameters were evaluated in urine and serum. Kidneys were harvested for histological examination and the assessment of crystal deposits.* Results*. In vitro and in vivo experiments demonstrated that treatment with* Glechoma longituba* extract significantly decreased calcium oxalate- (CaOx-) induced OPN expression, KIM-1 expression, and OS compared with the positive control group (*P* < 0.05). Additionally, in vivo rats that received* Glechoma longituba* extract exhibited significantly decreased CaOx deposits and pathological alterations (*P* < 0.05) compared with urolithic rats. Significantly lower levels of oxalate, creatinine, and urea and increased citrate levels were observed among rats that received* Glechoma longituba* (*P* < 0.05) compared with urolithic rats.* Conclusion*.* Glechoma longituba* has antiurolithic effects due to its possible combined effects of increasing antioxidant levels, decreasing urinary stone-forming constituents and urolithiasis-related protein expression, and elevating urinary citrate levels.

## 1. Introduction

Urolithiasis has afflicted humankind since antiquity and can lead to serious consequences throughout a patient's lifetime. Urolithiasis composed of calcium oxalate (CaOx), either alone or mixed with calcium phosphate, is hitherto the most common uroliths accounting for more than 80% of the stones. The mechanisms involved in the formation of CaOx stones are not fully understood. However, research on the pathogenesis of CaOx stone formation has indicated that the CaOx-induced generation of oxidative stress (OS) may be the initial trigger of a vicious cycle of urolithiasis formation [1, 2]. Excessive quantities of CaOx crystals could trigger the production of reactive oxygen species (ROS) in kidney tissues. ROS production leads to renal epithelial injury and apoptosis, which provide sites for crystal attachment and eventual retention within the kidneys [3, 4]. Furthermore, cell degradation following renal epithelial injury results in the generation of numerous membrane vesicles, which are effective crystal nucleators, enhance crystal nucleation at low supersaturation, and promote crystal-cell interactions [5]. Crystal attachment or retention will, in turn, induce the additional generation of OS.

Moreover, the high recurrence rate of CaOx urolithiasis remains an important unresolved problem. The 1-year recurrence rate in the majority of patients after extracorporeal shock wave lithotripsy and surgical intervention is approximately 10% [6]. Certain drugs, such as citrate and thiazide diuretic preparations, may have side effects that compromise their long-term use and are not consistently effective.

Many phytomedicine plants provide an inexpensive source of medicine for the majority of the world's population. Such medicines are considered safe and present minimal or no side effects, and studies [7–9] have indicated that numerous herbs, including* Viburnum opulus L.*,* Desmodium styracifolium*, and* Solanum xanthocarpum*, can be useful antiurolithic agents due to their antioxidant properties. In particular, flavonoids protect the kidney against the damaging effects of ROS, which may be the initial trigger of urolithiasis formation, as stated above. Our previous study suggested that catechin, an antioxidant, exhibited antinephrolithic potential by inhibiting ROS, apoptosis, phospho-P38 signaling, and OPN in rats [10].

The dried aerial part of* Glechoma longituba* (Nakai) Kupr (Labiatae) (referred to herein as* Glechoma longituba*) is distributed widely in Asia, Europe, and America and has been used for centuries in traditional Chinese medicine for the treatment of cholelithiasis, urolithiasis, dropsy, and various ailments, including asthma, bronchitis, cold, and inflammation [11, 12]. Previous research on the chemical constituents of this plant, including phenolic acid, flavonoids, triterpenoids, and essential oils, has led to the isolation and identification of these substances [13]. Pharmacological studies have indicated that phenolic compounds, such as caffeic acid, chlorogenic acid, and rosmarinic acid, have significant antioxidant bioactivities, whereas flavonoids are known for their antioxidant and antiradiation abilities [14–16].

Therefore, the present study was performed to determine the effects of* Glechoma longituba* on urolithiasis-related biochemical parameters and the antioxidative effects on calcium oxalate urolithiasis using in vitro and in vivo models and to compare the results to those with potassium citrate. This information is important in evaluating the potential of* Glechoma longituba* for the treatment of urolithiasis in a clinical setting.

## 2. Materials and Methods

### 2.1. Plant Materials, Extraction, and Fraction

*Glechoma longituba* was purchased from the Beijing Tongrentang Co., Ltd (Beijing, China) and graciously identified by members of the School of Pharmacy at Lanzhou University. A long-lasting decoction is used in traditional Chinese medicine, and the antioxidant levels from medicinal plants are not influenced by aqueous extraction; thus, the traditional decoction method was used to examine the effect of the aqueous extract [17]. Methods for extracting active ingredients from plants have been previously described. Briefly, 1,000 g of raw material was mixed with 5.0 L of distilled water for 60 min and decocted for approximately 25 min, and the supernatant was collected after filtration. Subsequently, 3.0 L of fresh distilled water was added to the residue, and the mixture was boiled. Finally, we combined the two aqueous extracts. After filtration, concentration, and lyophilization, the freeze-dried powder was stored at −80°C until use (yield = 51 g).

### 2.2. Cell Lines (HK-2) Experiment

The human kidney epithelial cell line HK-2, obtained from the School of Life Sciences at Lanzhou University, was maintained in subconfluent monolayers at 37°C in 5% CO_2_. The culture was grown and maintained in 25 cm^2^ Falcon tissue culture flasks as we described previously [18].

Cells were incubated with CaOx crystals (67 *μ*g/cm^2^) for 24 hours to establish a cell model [19]. Approximately 1 hour prior to CaOx exposure, the cells were treated with four different concentrations of* Glechoma longituba* (0.5, 1.0, 2.0, or 4.0 mg/mL) or 1.0 mg/mL potassium citrate (positive control). 24 hours after CaOx crystals exposure, the cells were washed three times with serum-free medium and harvested to assess their levels of SOD, MDA, CAT, OPN, and KIM-1.

### 2.3. Experimental Animals and Designs

Sixty male Sprague-Dawley (SD) rats, each weighing approximately 200 g, were purchased from the Animal Experimental Center of Lanzhou University and acclimated to a room at 25°C under a 12 h light, 12 h dark cycle. The rats were housed at the Institute of Urology of Lanzhou University Second Hospital (Lanzhou, Gansu) and fed a standard commercial rat chow. After acclimatization for 1 week, the rats were divided into six study groups of 10 animals each. Group 1 was used as the control group. The rats in the experimental groups (groups 2, 3, 4, 5, and 6) were given free access to drinking water containing 1.0% ethylene glycol as a stone inducer throughout the entire 4-week experimental period [20]. The rats in group 2 were treated as the urolithic group; the rats in group 3 were treated with potassium citrate at 2.0 g/kg/day for 28 days; the rats in group 4 were fed daily with* Glechoma longituba* at a dose of 220 mg/kg/day using oral gavage; and the rats in group 5 were fed daily with* Glechoma longituba* at a dose of 440 mg/kg/day using oral gavage; the rats in group 6 were fed daily with* Glechoma longituba* at a dose of 880 mg/kg/day using oral gavage.

### 2.4. Analysis of Serum and Urine

Urine samples were collected over 24 h at the end of the experiment by placing the rats individually in metabolic cages. Food and water were given to the rats during the 24 h urine collection period, and serum was also obtained for a biochemical analysis at the end of the experiment. We drew blood samples from the tails of the rats, and parameters for the known risk factors of stone disease, such as urine calcium, phosphate, and pH values, were measured with a Hitachi-7150 and Roche OMNI C analyser. Oxalate and citrate were measured using commercially available kits (BioVision Ltd., Milpitas, CA, USA; Instruchemie Ltd., Netherlands). Blood creatinine and urea levels were also measured. Malondialdehyde (MDA), superoxide dismutase (SOD), and catalase (CAT) in the blood and kidneys were measured using three kits (Jiancheng Bioengineering Ltd., Nanjing, China).

### 2.5. Analysis of OS

A cell lysis buffer was used to lyse cells for approximately 30 min at room temperature, and the lysates were collected to measure the levels of SOD, CAT, and MDA. A 0.1 g sample of renal tissue was mixed with 9 volumes of cold phosphate buffer (pH 7.4) and then homogenized. The homogenates were centrifuged at 16,000 ×g for 5 min at 4°C. The supernatants were measured for SOD, CAT, and MDA using the above kits.

### 2.6. Pathological Changes

The pathological alterations were graded as follows: 0: no visible lesions, 2: mild dilation of tubules, tubulointerstitial inflammatory infiltration, and lesion area < 20%; 4: dilation of tubules, tubulointerstitial inflammatory infiltration, and lesion area < 40%; and 6: severe dilation of tubules, massive tubulointerstitial inflammatory infiltration, and lesion area > 40% [21].

### 2.7. Renal Crystal Deposits

Crystal deposits in the kidney were evaluated as follows: no deposits: 0 points; crystal deposits in the papillary tip: 1 point; crystal deposits in the corticomedullary junction: 2 points; and crystal deposits in the cortex: 3 points. If crystal deposits were observed at multiple sites, the points were combined to provide a total score for each pathological section.

### 2.8. Immunohistochemical Staining

After preservation, the kidney sections were boiled for antigen retrieval and treated with 3% H_2_O_2_ to remove endogenous peroxidases. After rinsing with PBS, goat serum was used to block the sections. The sections were incubated at 4°C overnight with a polyclonal OPN antibody (rabbit polyclonal to OPN, ab8448, Abcam, Cambridge, UK) (5 *μ*g/mL) or a polyclonal KIM-1 antibody (rabbit polyclonal to KIM-1, Hopebiot) (1 : 500). After rinsing with PBS, the sections were incubated at 37°C with a polymer helper for 20 min. After rinsing three times with PBS, the slides were treated with poly-peroxidase-anti-rabbit/mouse IgG for 2 h before a final wash with PBS and staining with DAB.

### 2.9. Western Blot

Cells were washed twice with ice-cold PBS and then incubated in protein lysis buffer containing the protease inhibitor phenylmethylsulfonyl fluoride (PMSF) for 30 min. Then, cells were mechanically disrupted and centrifuged at 12,000 rpm for 15 min at 4°C to remove the nuclei.

The kidney tissue (100 mg) was homogenized in 1 mL of lysis buffer mixed with 10 *μ*L of phenylmethanesulfonyl fluoride (100 mM) before centrifugation (12,000 ×g for 5 min) at 4°C. The supernatants were collected to determine the protein concentration. For the Western blot analysis, 80 *μ*g of protein was separated using SDS-PAGE and transferred to polyvinylidene difluoride (PVDF) membranes, which were then blocked in 5% fat-free milk at room temperature for 2 h. Following incubation with OPN or KIM-1 antibody at 4°C overnight and three washes in 1x Tris-buffered saline for 15 min, the PVDF membrane was then incubated with antibodies labeled with horseradish peroxidase at room temperature for 1 h. The membranes were washed three times for 5 min each with 1x Tris-buffered saline, and the proteins were detected via enhanced chemiluminescence. The GAPDH antibody served as a control for protein loading.

### 2.10. Statistical Analyses

The results were presented as the means (SD). A one-way ANOVA followed by Tukey's post hoc multiple comparison test was used to perform comparisons between the groups. SPSS version 18 was used for the analyses, and *P* < 0.05 was considered significant.

## 3. Results

### 3.1. Changes in SOD, MDA, CAT, OPN, and KIM-1 Expression Levels in HK-2 Cells

The cellular SOD and CAT levels decreased and the MDA levels were upregulated after exposure to 66 *μ*g/cm^2^ of CaOx for 24 hours compared with untreated cells (*P* < 0.05) (Table 1). However, these indicators were not improved after treatment with potassium citrate. After pretreatment with* Glechoma longituba* at the concentration of 2.0 mg/mL, the MDA significantly decreased, and the SOD and CAT levels significantly increased compared with cells exposed to 66 *μ*g/cm^2^ of CaOx for 24 hours. Additionally, the treatment of* Glechoma longituba* at a concentration of 4.0 mg/mL nearly improved the CaOx-induced OS to normal levels.

**Figure 1:**
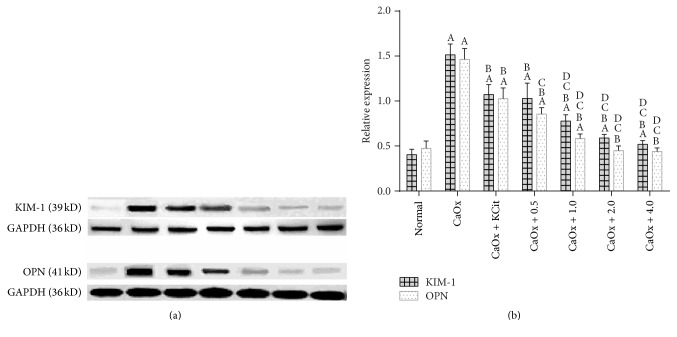
Effects of potassium citrate and* Glechoma longituba* on CaOx-induced HK-2 cell protein expression (^A^ *P* < 0.05 compared with normal group; ^B^ *P* < 0.05 compared with CaOx group; ^C^ *P* < 0.05 compared with CaOx + KCit group; and ^D^ *P* < 0.05 compared with CaOx + 0.5 group, *n* = 5).

Western blot was used to detect the expression of OPN and KIM-1 in the cells. When the cells were exposed to CaOx, KIM-1 expressions were upregulated (*P* < 0.05), which were further reduced by potassium citrate and* Glechoma longituba* at concentrations from 0.5 to 1.0, 2.0, or 4.0 mg/mL (*P* < 0.05) (Figure 1(b)). Additionally, compared with the group pretreated with potassium citrate, the KIM-1 expressions significantly decreased after pretreatment with* Glechoma longituba* at the concentration of 1.0 to 4.0 mg/mL. The quantitative analysis of OPN expression was consistent with the KIM-1 expression.

### 3.2. Urinary Variables in Different Groups

As shown in Table 2, at the end of the experimental period, the blood creatinine and urea levels in group 2 were significantly increased compared with that of group 1 (*P* < 0.05). However, the blood creatinine and urea in group 3 were significantly decreased after 2 g/kg/day potassium citrate administration compared with that for group 2 (*P* < 0.05). Additionally, the above two indicators in groups 4, 5, and 6 were significantly decreased after* Glechoma longituba* administration compared with that of group 2 (*P* < 0.05).

The urine pH was significantly increased in group 3 compared with group 2 (*P* < 0.05). However, the urine pH was not significantly improved in group 4, group 5, or group 6 compared with group 2. The urine oxalate levels were significantly increased in group 2 and subsequently reduced with* Glechoma longituba* administration, which suggested the potential oxalate reduction capabilities of* Glechoma longituba*. Additionally, urine oxalate was significantly decreased in groups 5 and 6 compared with group 4 (*P* < 0.05), and urine citrate was significantly increased in groups 3, 5, and 6 compared with group 2 (*P* < 0.05).

### 3.3. Antioxidant Effect of* Glechoma longituba* Extract

**Figure 2:**
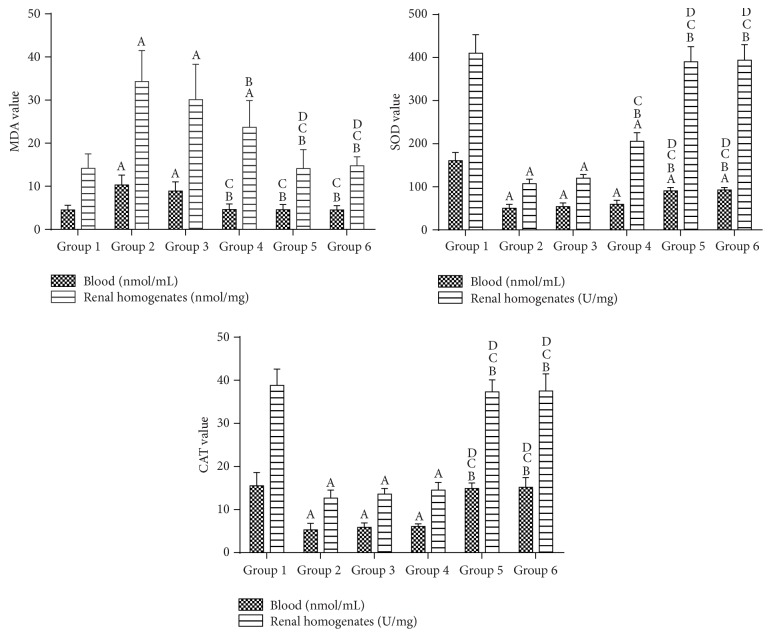
Activities of MDA, SOD, and CAT in the blood and renal homogenates. The values shown are the means ± SD. ^A^ *P* < 0.05 compared with group 1; ^B^ *P* < 0.05 compared with group 2; ^C^ *P* < 0.05 compared with group 3; and ^D^ *P* < 0.05 compared with group 4.

Figure 2 shows the activities of MDA, SOD, and CAT in the blood and kidney homogenates. Compared with group 1, the MDA values in group 2 increased significantly and the SOD and CAT values decreased significantly in the blood and kidney samples (*P* < 0.05). However, the activities of MDA, SOD, and CAT in group 3 did not significantly improve in the blood or kidney samples compared with that of group 2 (*P* > 0.05).

Compared with group 2, the MDA values in groups 4, 5, and 6 significantly decreased in both the blood and kidney samples (*P* < 0.05), and the MDA values in groups 5 and 6 decreased significantly in the kidney samples compared with that of group 4. Moreover, the renal SOD levels in group 4 and the blood and renal SOD levels in groups 5 and 6 were significantly improved compared with those of group 2 (*P* < 0.05). The blood SOD levels were slightly improved in group 4, although not to a significant extent. The SOD levels were significantly increased in groups 5 and 6 in both the blood and kidney samples compared with that of group 4 (*P* < 0.05). Moreover, compared with group 2, the CAT levels in group 5 and 6 improved significantly in both the blood and kidney samples (*P* < 0.05).

Thus, treatment with* Glechoma longituba* extracts protected against the changes associated with oxidative stress.

### 3.4. Pathological Changes

**Figure 3:**
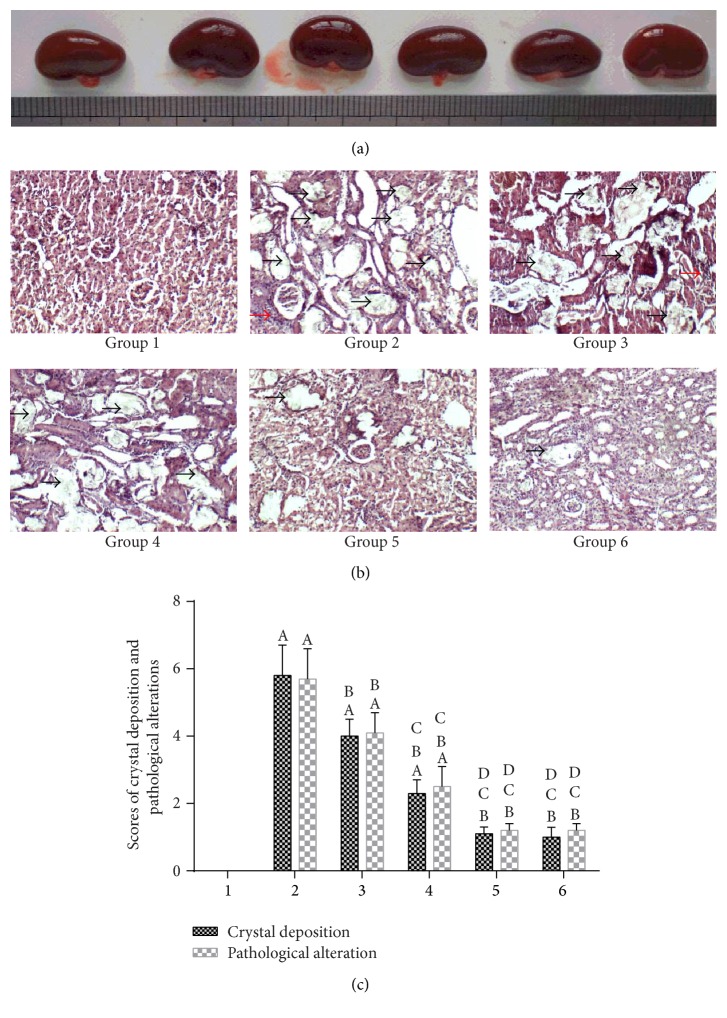
Histopathological view and crystal deposition in the kidneys of rats. (a) Gross anatomy of the kidney (from left to right are groups 1 to 6, resp.). (b) Micrograph of renal tissue with HE staining. (c) Scores for pathological alterations and crystal deposition. Black arrows indicate renal crystals. Red arrows indicate inflammatory infiltration. The values shown are the means ± SD. ^A^ *P* < 0.05 compared with group 1; ^B^ *P* < 0.05 compared with group 2; ^C^ *P* < 0.05 compared with group 3; and ^D^ *P* < 0.05 compared with group 4.

Figure 3(a) shows that pathological changes, such as focal hemorrhage and severe swelling, were not found in the kidneys of group 2, which indicated that a stone inducer of 1.0% ethylene glycol throughout the entire 4-week experimental period could not lead to renal gross alterations. The gross alterations of the kidneys in groups 2 to 6 were similar to those of group 1.

Figure 3(b) shows that the kidney sections stained with HE in group 1 revealed the normalities in different segments of the nephrons. Additionally, considerable tubulointerstitial changes, such as tubular atrophy, dilation, hyaline cast, tubular cell necrosis, and interstitial inflammation (red arrow), were observed in the renal tissue in group 2. Tubulointerstitial damage was significantly decreased in groups 3, 4, 5, and 6 compared with group 2 (*P* < 0.05), and these pathological changes in groups 4, 5, and 6 were significantly improved compared with those in group 3 (*P* < 0.05) (Figure 3(c)).

### 3.5. Renal Crystal Deposits

As shown in Figure 3, the amount of birefringent crystalline deposits (black arrow) from the histological slices of the tubules of all regions, including the cortex, medulla, and papilla, was observed in all of the animals in group 2. However, in groups 3, 4, 5, and 6, a lower number of CaOx deposits were found inside the proximal tubule, loop of Henle, distal tubule, and collecting duct compared with that of group 2 (*P* < 0.05). Moreover, the number of CaOx deposits in groups 4, 5, and 6 was significantly decreased compared with that of group 3; significant differences were observed between (1) groups of 5 and 6 and (2) group 4.

### 3.6. Immunohistochemical Staining

**Figure 4:**
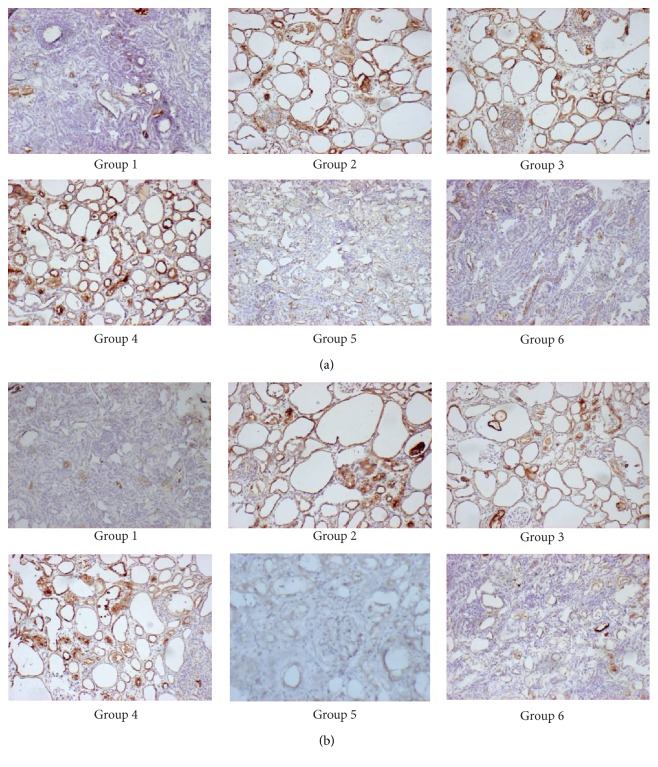
Immunohistochemistry of KIM-1 (a) and OPN (b) protein expression in the kidneys. Group 1: control group; group 2: urolithiasis group; and group 3: potassium citrate-treated urolithiasis group.* Glechoma longituba* was gavaged 220 mg/kg/day, 440 mg/kg/day, and 880 mg/kg/day to groups 4, 5, and 6 via oral tube feeding, respectively (100x).

The immunohistochemical staining results showed that KIM-1 protein expression was weak in the renal tissues of group 1 (Figure 4(a)). The renal cortex of group 2 showed a high frequency and intensity of staining for KIM-1 in the proximal tubule, distal tubule, and collecting duct. The intensity of staining was greater with crystal deposition, particularly in the proximal tubules, where the crystals were positive for KIM-1 (Figure 4(a)). Group 3 also showed a clear expression of KIM-1 protein (Figure 4(a)). However, in groups 5 and 6, KIM-1 expression was faint (Figure 4(a)). The OPN expression in all groups was similar to that of the KIM-1 protein (Figure 4(b)).

### 3.7. Western Blot

**Figure 5:**
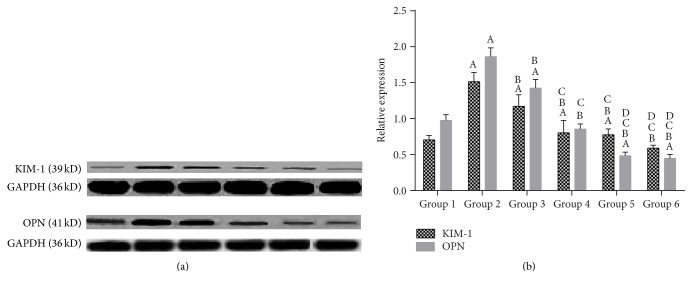
Analysis of KIM-1 and OPN protein expression in the SD rat kidneys using conventional Western blot. GAPDH from the samples was designated as the internal control. The columns and bars represent the means ± SD (^A^ *P* < 0.05 compared with group 1; ^B^ *P* < 0.05 compared with group 2; ^C^ *P* < 0.05 compared with group 3; and ^D^ *P* < 0.05 compared with group 4).

Western blot assays were performed to detect the expression of KIM-1 and OPN in the rat kidneys. The KIM-1 protein was concentrated as a distinct band at 39 kD in group 2 and the potassium citrate-treated urolithic group. In the control groups and the* Glechoma longituba*-treated urolithic group, only faint bands were observed (Figure 5(a)). Figure 5(a) shows the GAPDH expression in the corresponding samples. The quantitative analysis of OPN expression was consistent with that of KIM-1 expression, with group 2 exhibiting the highest concentrations; the potassium citrate-treated urolithic group showed lower expression than group 2, and group 6 showed the lowest expression (Figure 5(b)).

## 4. Discussion

Many medicinal plants are used to treat urolithiasis worldwide [17, 22, 23]. Plants provide an inexpensive source of medicine for the majority of the world's population, and such medicines are considered safe and present minimal or no side effects [24]. To investigate the medicinal applicability of* Glechoma longituba* in urolithiasis, we evaluated an extract for its antiurolithic activity using an in vitro assay and an in vivo rat model of urolithiasis.

Cell and rat studies have indicated that CaOx crystals could cause injury to renal cells [25, 26], most likely through the production of reactive oxygen species (ROS). Epithelial injury is considered a risk factor for crystallization and crystal deposition in the kidney because it promotes crystal nucleation, aggregation, retention, and stone development [25, 27]. Antioxidants, such as vitamin E, catechin, and selenium, have been shown to provide protection against oxidative injury via crystal deposition [28]. When tested for* Glechoma longituba* activity, these results showed that the protective effect of* Glechoma longituba* on renal epithelial cell may be due to its antioxidant activity because pretreatment with the* Glechoma longituba* significantly reduced the OS levels in cells and rats when exposed to CaOx crystals. The exact mechanism(s) by which* Glechoma longituba* exerts its antioxidant effects against ethylene glycol- (EG-) induced urolithiasis is not yet understood; it may be related to the flavonoids in the extract, which protect the kidney against the damaging effects of ROS, including peroxynitrite, peroxyl radicals, superoxide, singlet oxygen, and hydroxyl radicals [29].

Additionally, renal epithelial cells respond to CaOx crystals characteristically by increasing the expression of specific genes that encode transcriptional activators, extracellular matrix regulators, and growth factors and by the production of pro- and anti-inflammatory molecules. OPN and KIM-1 are modulators of biomineralization processes [4]. We observed that* Glechoma longituba* could regulate the urolithiasis-related protein OPN and KIM-1 expression in cell and rat experiments. OPN plays a major role in the adhesion function of calcium oxalate crystals by forming a major component of the extracellular matrix proteins [30]. In the present study, an increase in the OPN protein was observed in cells and kidneys after exposure to CaOx crystals. In previous reports, immobilized OPN increased crystal aggregation and OPN adherence to the surface of collagen granules, which can cause an increase in calcium oxalate crystal adherence and aggregation [31]. Thus, any molecule that can modulate renal OPN expression can be expected to regulate the aggregation and retention of calcium oxalate crystals in tissue.

KIM-1 levels are considered a sensitive biomarker of renal tubular injury [32, 33]; moreover, KIM-1 is conserved across species and upregulated after renal injury in zebrafish, mice, rats, nonhuman primates, and humans. In our study, the KIM-1 protein was also increased in cells and rats after treatment with CaOx as determined via Western blot analysis and immunohistochemical staining. However, the high levels of CaOx-induced protein of KIM-1 and OPN were further inhibited by potassium citrate and* Glechoma longituba* at different concentrations (*P* < 0.05). Additionally, the in vitro and in vivo experiments showed that the expressions of the two proteins significantly decreased after treatment with highest concentration of* Glechoma longituba*; this was in contrast with the group treated with potassium citrate.

Administering EG to rats is the most common and effective method for inducing CaOx renal crystals in vivo, and our present study successfully induced crystal formation using this model [34, 35]. The oral doses of* Glechoma longituba* were selected based on the clinical dosage provided by the Chinese Pharmacopoeia and the ratio of body to surface area between humans and rats. Hepatic enzymes could metabolize EG to oxalic acid, which combines with calcium ions in the renal tubule epithelium. Several biochemical abnormalities, including increased urine output, hyperoxaluria, hypomagnesiuria, hyperuricosuria, hypercalciuria, and hypocitraturia, have been observed in the urine of lithic animals [36]. In the present study, we found that EG could increase oxalate (the urinary stone-forming constituent) contents and decrease renal function; these findings are consistent with previous results. However, hypercalciuria was not observed in group 2 as previously described. A possible explanation for this result is that the formation of CaOx consumes free calcium (Ca) and normalizes Ca levels in the urine.

Potassium citrate (KCit) was approved in the United States by the Food and Drug Administration in 1985 to treat a wide variety of disorders that cause stone formation. Citrate is an important inhibitory substance in the urinary tract that forms soluble complexes with calcium, reduces the saturation of calcium phosphate and calcium oxalate, and inhibits crystallization and kidney stone enlargement. Therefore, citrate decreases the formation of calcium-containing stones [37]. KCit has been demonstrated to be useful for controlling uric acid stones and calcareous stones by urinary alkalinization and by increasing urinary citrate, respectively [38]. Our experiments indicated that urine citrate and pH levels were significantly increased in group 3 compared with those of group 2. KCit has been proven to be useful for controlling calcareous stones by increasing urinary citrate; thus, in the present study, KCit appears to have produced an antiurolithic effect mainly by increasing urinary citrate rather than by raising urinary pH. Moreover, we found that, in group 3, EG-induced crystal deposition and pathological changes were inhibited; in addition, renal function was improved.

After oral treatment with the* Glechoma longituba* aqueous extract, we observed an improvement in urinary oxalate, citrate, renal depositions, and renal function compared with that of the EG-induced rats and KCit-treated rats.

It is not uncommon for plant extracts to interfere with oxalate metabolism in animal models of urolithiasis. For instance, an extract of* Aerva lanata* decreases oxalate excretion in ethylene glycol-fed rats by decreasing the formation of oxalate-synthesizing enzymes such as glycolic acid oxidase in the liver and lactate dehydrogenase in the liver and kidneys [39]; similar results were obtained for an extract of* Tribulus terrestris* [40]. Thus, we speculate that, similar to the two aforementioned extracts,* Glechoma longituba* aqueous extract may contain certain compounds that decrease oxalate excretion by inhibiting the formation of oxalate-synthesizing enzymes. In fact, we are unaware of any study in which components of* Glechoma longituba* aqueous extract have been reported to decrease the formation of oxalate-synthesizing enzymes, and future research is required to clarify this point. However, it must be recognized that, under in vivo conditions,* Glechoma longituba* could decrease oxalate, which may combine with calcium ions in the renal tubule epithelium, potentially leading to the growth of crystals and ultimately the formation of stones.

Additionally, the KCit-treated rats had higher crystal deposits, OPN and KIM-1 expression levels, and creatinine and urea levels relative to the* Glechoma longituba*-treated rats, which presented nearly normal histologies, crystal deposits levels, protein expressions, and renal function. The renal ROS levels and urinary oxalate levels of the KCit-treated rats were also high despite the administration of citrate. These results suggest that* Glechoma longituba* can be considered a better antiurolithic agent than potassium citrate. To the best of our knowledge, this is the first evidence supporting the inhibitory effect of* Glechoma longituba* on CaOx crystal formation in EG-fed rats.

## 5. Conclusions

In conclusion, our results indicate that the extract prevents the deposition of CaOx crystals in the rat kidney, possibly by increasing antioxidant levels, decreasing urinary stone-forming constituents and urolithiasis-related protein expression, and elevating urinary citrate levels. Moreover, treatment with* Glechoma longituba* was found to be more effective than the potassium citrate therapy used in the clinical management of urolithiasis. Hence,* Glechoma longituba* offers promising potential as an antiurolithic agent. Further studies are necessary to identify the active components in the extract and the mechanisms responsible for the observed pharmacological activity.

## Figures and Tables

**Table 1:** Effect of *Glechoma longituba* extracts on OS parameters of HK-2 cells ($\overset{-}{x}\pm s$).

Parameter (units)	Normal	CaOx	CaOx + KCit	CaOx + 0.5	CaOx + 1.0	CaOx + 2.0	CaOx + 4.0

CAT (NU/mL)	21.4 ± 4.29	5.1 ± 1.06^a^	4.9 ± 0.88^a^	5.6 ± 0.52^a^	5.7 ± 0.89^a^	12.6 ± 1.78^abcd^	19.8 ± 2.34^bcd^
SOD (NU/mL)	38.7 ± 6.5	8.5 ± 1.83^a^	8.1 ± 1.18^a^	10.3 ± 2.16^a^	19.7 ± 4.67^abcd^	26.5 ± 4.01^abcd^	34.9 ± 6.21^bcd^
MDA (nmol/mL)	8.9 ± 0.75	32.5 ± 5.89^a^	34.4 ± 4.98^a^	29.4 ± 3.80^a^	19.7 ± 3.12^abcd^	10.5 ± 1.56^bcd^	9.1 ± 1.33^bcd^

Normal: HK-2 cells were treated with medium only. CaOx: the cells were incubated with CaOx crystals (67 *μ*g/cm^2^) for 24 hours to establish a cell model. CaOx + KCit: approximately 1 hour prior to CaOx exposure, cells were treated with 1.0 mg/mL potassium citrate (positive control). CaOx + 0.5, CaOx + 1.0, CaOx + 2.0, and CaOx + 4.0: approximately 1 hour prior to CaOx exposure, cells were treated with four different concentrations of *Glechoma longituba* (0.5, 1.0, 2.0, or 4.0 mg/mL, resp.). Values are expressed as means ± SD. (^a^ *P* < 0.05 compared with normal group; ^b^ *P* < 0.05 compared with CaOx group; ^c^ *P* < 0.05 compared with CaOx + KCit group; and ^d^ *P* < 0.05 compared with CaOx + 0.5 group, *n* = 5).

**Table 2:** Effect of *Glechoma longituba* extracts on blood and urine biochemical parameters of SD rats ($\overset{-}{x}\pm s$).

Parameter (units)	Group 1	Group 2	Group 3	Group 4	Group 5	Group 6
Blood plasma parameters						
Creatinine (mmol/L)	28.74 ± 6.5	78.31 ± 10.91^a^	31.16 ± 7.18^b^	30.39 ± 8.06^b^	29.96 ± 6.79^b^	29.85 ± 7.48^b^
Urea (*μ*mol/L)	7.86 ± 1.82	22.68 ± 4.66^a^	8.89 ± 1.96^b^	7.79 ± 2.78^b^	8.40 ± 1.69^b^	7.87 ± 1.71^b^
Parameters in urine pH	6.53 ± 0.87	6.44 ± 0.54	8.11 ± 1.24^ab^	6.35 ± 0.64^c^	6.40 ± 0.73^c^	6.59 ± 0.93^c^
Calcium (mmol/L)	3.75 ± 1.31	3.55 ± 0.21	3.68 ± 0.98	3.57 ± 1.02	3.53 ± 1.41	3.40 ± 1.02
Oxalate (*μ*mol/L)	92.22 ± 20.12	490.97 ± 89.64^a^	477.26 ± 78.64^a^	170.58 ± 30.73^abc^	89.14 ± 15.18^bcd^	88.76 ± 18.92^bcd^
Phosphate (mmol/L)	26.13 ± 7.82	27.01 ± 11.43	29.55 ± 13.7	26.61 ± 9.5	30.64 ± 12.5	29.85 ± 11.46
Citrate (mmol/L)	0.96 ± 0.27	0.94 ± 0.41	4.29 ± 1.55^ab^	1.02 ± 0.39^c^	2.01 ± 0.65^abcd^	1.98 ± 0.45^abcd^

Values are expressed as means ± SD. (^a^ *P* < 0.05 compared with group 1; ^b^ *P* < 0.05 compared with group 2; ^c^ *P* < 0.05 compared with group 3; and ^d^ *P* < 0.05 compared with group 4; *n* = 10).

## Abbreviations

CaOx: Calcium oxalate
EG: Ethylene glycol
OS: Oxidative stress
SD: Sprague-Dawley
SOD: Superoxide dismutase
ROS: Reactive oxygen species
MDA: Malondialdehyde
CAT: Catalase
OPN: Osteopontin
KIM-1: Kidney injury molecule-1
BUN: Blood urea nitrogen
Cr: Serum creatinine.
